# Application of Muscle Thickness and Quality Measured by Ultrasound in Frailty Assessment in China

**DOI:** 10.3389/fmed.2022.859555

**Published:** 2022-03-31

**Authors:** Shan Lv, Ling Ling, Hui Shi, Xing Chen, Shu Chen, Siping Zhu, Wei Lin, Rong Lv, Guoxian Ding

**Affiliations:** ^1^Department of Geriatrics, Jiangsu Province Hospital and The First Affiliated Hospital With Nanjing Medical University, Nanjing, China; ^2^Department of Gerontology, Suzhou Ninth People's Hospital, Suzhou, China; ^3^NHC Contraceptives Adverse Reaction Surveillance Center, Jiangsu Health Development Research Center, Nanjing, China

**Keywords:** Fried Frailty Phenotype (FFP), ultrasound, muscle thickness (MT), muscle quality, local muscle

## Abstract

To explore the correlation between Fried Frailty Phenotype (FFP) and the muscle thickness and quality of local muscle, and to provide a reasonable basis for the application of ultrasound measurement in the frailty assessment. A total of 150 people (age ≥ 65 years, 58 women, 92 men) were included from the First Hospital Affiliated to Nanjing Medical University. They were divided into Normal group (40 cases), Prefrailty group (69 cases) and Frailty group (41 cases). The thickness and the quality of local muscle were detected by ultrasound. Participants in the prefrailty group had a higher grayscale value of the vastus lateralis muscle, indicating the deterioration of muscle quality. At the frailty stage, the muscle thickness and quality of the vastus lateralis muscle and the anterior tibialis muscle decreased significantly compared with the normal and the prefrailty group. Pearson's correlation analysis also showed FFP was negatively correlated with muscle thickness and quality of the lower limbs. In multiple regression model, FFP was positively associated with gray value (Vastus lateralis muscle:β =0.457, *p* < 0.001; Anterior tibialis muscle: β = 0.220, *p* = 0.037) and inversely associated with muscle thickness (Vastus lateralis muscle:β = −0.973, *p* = 0.031; Anterior tibialis muscle: β = −4.551, *p* = 0.004) in the frailty stage. Together, FFP was closely related to muscle thickness and quality, especially vastus lateralis muscle. Moreover, Muscle quality has deteriorated in the prefrailty stage, which is earlier than muscle thickness.

## Key Points

1. A rapid and accurate screening test is needed to assess frailty, as current assessments are complex and time-consuming.
2. Ultrasound can measure muscle thickness and quality simultaneously.
3. Fried Frailty Phenotype (FFP) was closely related to muscle thickness and quality, especially vastus lateralis muscle.
4. Muscle quality has deteriorated in the stage of prefrailty, which is earlier than muscle thickness.
5. Our findings highlight the practicability of ultrasonic measures of local muscle with frailty assessment.

## Introduction

With the global aging and the improvement of people's living standards, frailty has gradually become a research hotspot in the field of geriatrics (1). Frailty is caused by a variety of factors, a clinical syndrome characterized by reduced strength, stamina, and reduced physical function that causes increased fragility in the individual and ultimately leads to fall, disability, and/or death (2). So far, various methods of frailty assessment have been used, but there is no unified standard and they are all complicated and time-consuming (3, 4).

Sarcopenia is often considered as an early manifestation of frailty, an important risk factor for accelerating the occurrence and development of frailty, and a core element of frailty (5). With the deepening of the research on sarcopenia, it has been reported that the speed of sarcopenia caused by aging is not consistent in all regions of the body (6). As a new tool for the evaluation of sarcopenia, ultrasound has the advantage of accurately evaluating the local muscle thickness of the body and the muscle quality simultaneously (7, 8). Muscle quality refers to the micro and macro changes in muscle structure and composition, as well as the muscle function transmitted per unit in muscle mass (9). At present, several studies have shown that the changes of muscle mass and muscle strength are inconsistent, indicating muscle quality may play an important role and is a reliable indicator of muscle strength (10, 11).

However, the relationship between local muscle thickness, muscle quality and frailty assessment is not clear. Therefore, we selected the same part of the anterior ulnar muscle and the vastus lateral muscle and anterior tibia muscle to detect their muscle thickness by ultrasound. Meanwhile, QLab software was used to analyze the gray value of the Region Of Interest (ROI). The purpose of this study was to investigate the correlation between Fried frailty assessment and the muscle thickness and muscle quality of anterior ulnar muscle, vastus lateralis muscle and anterior tibial muscle, and to provide a reasonable basis for the application of ultrasound measurement in the assessment of frailty.

## Materials and Methods

### Study Participants

Data of patients hospitalized in the Department of Geriatrics Endocrinology from January 2020 to January 2021 were collected. A total of 150 inpatients were included in this study, including 92 men and 58 women. Their inclusion criteria were: (1) they were older than 65 years old; (2) they had the ability of independent activities and were in good general condition. Exclusion criteria:(1) patients with autoimmune diseases, musculoskeletal diseases, or thyroid dysfunction; (2) Patients with severe heart, liver and kidney function impairment or with tumor, severe infection and other diseases; (3) recent operation or serious external injury; (4) People who cannot move autonomously and suffer from mental illness; (5) exclude other endocrine diseases, such as the pituitary, adrenal, parathyroid and other diseases; (6) take sex hormones, glucocorticoids, thyroid hormones, antiepileptic drugs, antidepressants and other drugs that affect muscle metabolism. This clinical study was approved by the Ethics Committee of the First Affiliated Hospital of Nanjing Medical University (No. 2019-SR-481).

### Biochemical Analysis

Height and weight were measured by standard methods with participants wearing light clothing without shoes. Body mass index (BMI) was calculated as BMI (kg/m^2^) = Weight (kg)/height^2^ (m^2^).

After overnight fasting, blood samples of participants were obtained and centrifuged at 4.0°C for 10 min at 1,000 rpm and subsequently analyzed. Plasma glucose was determined using the YSI 2300 STAT Plus glucose oxidase assay (Yellow Springs Instruments, Yellow Springs, OH, USA). Serum insulin was measured using a radioimmunoassay (EMD Millipore, Billerica, MA, USA). Serum triglycerides (TG) and cholesterol were analyzed using enzymatic methods with an automated platform (Roche Modular Diagnostics, Indianapolis, IN, USA). Serum triiodothyronine (FT3), thyroxine (FT4), and thyroid stimulating hormone (TSH) levels were measured using the Abbott AxSYM Immunoassay system (Abbott Laboratories, Abbott Park, IL, USA) with intra- and inter-assay coefficients of variation of <10% for all measurements.

### Ultrasound Measurements

A B-mode ultrasound (Philips iU Elite, Bothell, WA, USA) with a linear transducer (5–12 MHz) was used to evaluate muscle thickness (MT) and muscle quality. When measuring the anterior ulnar muscle, MT was obtained on the anterior of the right forearm (at 30% proximal between the styloid process and the head of the radius) when the participants were in the supine position with their elbow extended, relaxed and their forearm supinated. When measuring the vastus lateralis muscle, the probe was placed at the junction of the greater trochanter of femur and the middle and lower third of the femur, with the long axis perpendicular to the long axis of femur, and the maximum thickness of the vastus lateralis muscle was measured. When measuring the anterior tibialis muscle, the probe was placed at the midpoint of the horizontal line between the lower edge of the patella and the lateral condyle of the fibula. The long axis of the probe was perpendicular to the long axis of the fibula to measure the thickest part of the anterior tibial muscle.

When measuring the muscle quality, select the same part of the muscle, adjust the inspection depth to 5 cm, freeze the image, analyze it with QLAB software, use the default 5 mm square sampling frame, avoid the blood vessels, obtain the region of interest (ROI), and then measure the gray value of ROI.

All measurements were performed by the same sonographer with 5 years of experience. All data were measured three times, and the average value was used for further analysis.

### Depression and Nutritional Assessment

The severity of depressive mood was evaluated using the 30-item Geriatric Depression Scale (GDS-30) developed by Yesavage et al. (12). The GDS-30 was rater-administered in a standardized manner. All items in the GDS-30 are rated as 0 or 1; specifically, 1 = “No” and 0 = “Yes” for some items (1, 5, 7, 9, 15, 19, 21, 27, 29, 30) but 0 = “Yes” and 1 = “No” for the remaining items. Item scores are summed, resulting in a possible total score of 0–30. High scores represent more severe depression.

Nutritional status was evaluated by Mini Nutritional Assessment (MNA) composed of simple measurements and brief questions. Discriminant analysis was used to compare the nutritional status determined by the extensive nutritional assessment including complete anthropometric, clinical biochemistry and dietary parameters. The sum of the MNA score distinguishes between elderly patients with: 1. Adequate nutritional status (MNA > or = 24); 2. Malnutrition (MNA < 17; 3); 3. At risk of malnutrition (MNA between 17 and 23.5).

### Frailty Measures

Frailty status was assessed at discharge with a modified version of the frailty phenotype by Fried (13) including unintentional weight loss, feelings of exhaustion, weakness (grip strength), gait speed, and independence in Activities of Daily Living (ADL) measured with the Katz Index (14). Weight loss and exhaustion relied on participant self-report. Grip strength was assessed by dynamometry, and walking speed was based on a 15-foot timed gait. Cut-off scores, as defined by Fried, were used for gait speed and grip strength.

The overall frailty status of the patient was assessed based on the above domains. Patients with problems in ≥ 3 domains were considered as frail and 1–2 domains were considered as pre-frail.

### Ethical and Legal Considerations

The participants themselves gave their written informed consent to participate in the study and were informed that they could refuse to participate at any stage.

### Statistical Analysis

Descriptive data are presented as the means ± SDs. The associations between Fried Frailty Phenotype (FFP) and muscle mass, muscle quality were examined using Pearson's correlation analysis. Comparison between multiple groups was performed by one-way ANOVA. Multivariate logistics regression analysis models were used to analyze muscle mass, muscle quality and the FFP using age, BMI data and so on as confounding variables. All statistical analyses were performed using SPSS V.20.0 (IBM Corp, Armonk, New York, USA), and *p* < 0.05 was considered statistically significant.

## Results

### General Characteristics and Fried Frailty Phenotype of Participants

Table 1 shows the participant's demographic characteristics. The analysis included data from 150 older inpatients, of whom 92 were men and 58 were women. According to Fried diagnostic criteria, the patients in the three groups were divided into three groups: normal group (FFP 0 points), pre-frailty group (FFP 1–2 points), and frailty group (FFP 3 points). The average age of the patients in the three groups was over 65 years old. The mean GDS-30 scores in the frailty and pre-frailty groups were 9.17 ± 5.16 and 6.38 ± 5.99 points, respectively. The MNA scores of the three groups were within the range of adequate nutritional status.

**Table 1:** Anthropometrics, depression assessment and Fried Frailty Phenotype (FFP) of the participants.

	**Normal**	**Prefrailty**	**Frailty**
***n***	**40**	**69**	**41**
Age (years)	72.77 ± 6.41	75.58 ± 8.32	85.38 ± 6.72^***^^###^
Weight (cm)	67.38 ± 6.74	66.27 ± 10.67	62.86 ± 12.20
Height (cm)	164.54 ± 8.26	164.71 ± 8.11	163.30 ± 7.21
BMI (kg/m2)	24.85 ± 3.27	24.44 ± 3.62	23.55 ± 4.33
HbA1c (%)	6.57 ± 1.20	6.69 ± 1.56	7.22 ± 2.40^*^
Glucose (mmol/L)	5.93 ± 1.51	5.78 ± 1.46	6.70 ± 2.43^*^
TC (mmol/L)	4.43 ± 1.24	4.42 ± 1.14	4.24 ± 1.13
TG (mmol/L)	1.32 ± 0.51	1.45 ± 0.69	1.32 ± 0.73
HDL-C (mmol/L)	1.23 ± 0.25	1.16 ± 0.32	1.14 ± 0.32
LDL-C (mmol/L)	2.57 ± 0.82	2.62 ± 0.83	2.51 ± 0.74
VD (ng/ml)	66.54 ± 24.02	52.42 ± 25.95^*^	48.65 ± 17.85^***^
FT3 (pmol/L)	4.0.70 ± 1.27	4.34 ± 0.82	3.76 ± 0.58^***^^##^
FT4 (pmol/L)	15.97 ± 2.01	16.34 ± 2.06	16.59 ± 3.18
TSH (mIU/L)	2.49 ± 1.51	2.64 ± 1.42	2.88 ± 2.20
Depression	5.07 ± 6.63	6.38 ± 5.99	9.17 ± 5.16^**^
MNA	29.88 ± 3.57	28.42 ± 4.39	28.33 ± 5.42
FFP	0	1.65 ± 0.48^***^	3.62 ± 0.50^***^^###^

*Variables are expressed as mean ± SD; BMI, body mass index; HAc1%, glycated hemoglobin; TC, cholesterol; TG, triglyceride; HDL-C, high-density lipoprotein cholesterol; LDL-C, low-density lipoprotein cholesterol; FT3, triiodothyronine; FT4, thyroxine; TSH, thyroid stimulating hormone; MNA, Mini Nutritional Assessment; FFP, Fried Frailty Phenotype*.

*compared with normal;

*#compared with prefrailty*.

* *P < 0.05*;

** *and*

## *P < 0.01*;

*** *and*

### *P < 0.001*.

The age of the frailty group was significantly higher than that of the normal group. Compared with the normal group, weight, height, body mass index, lipid levels, TSH, MNA score of the frailty group and the pre-frailty group were not statistically significant. Vitamin D and FT3 in the frailty group were lower than those in the normal group. The HbA1c, fasting plasma glucose and depressed mood were higher than those of the normal group.

### Local Muscle Thickness and Muscle Quality of Upper and Lower Extremities

**Figure 1:**
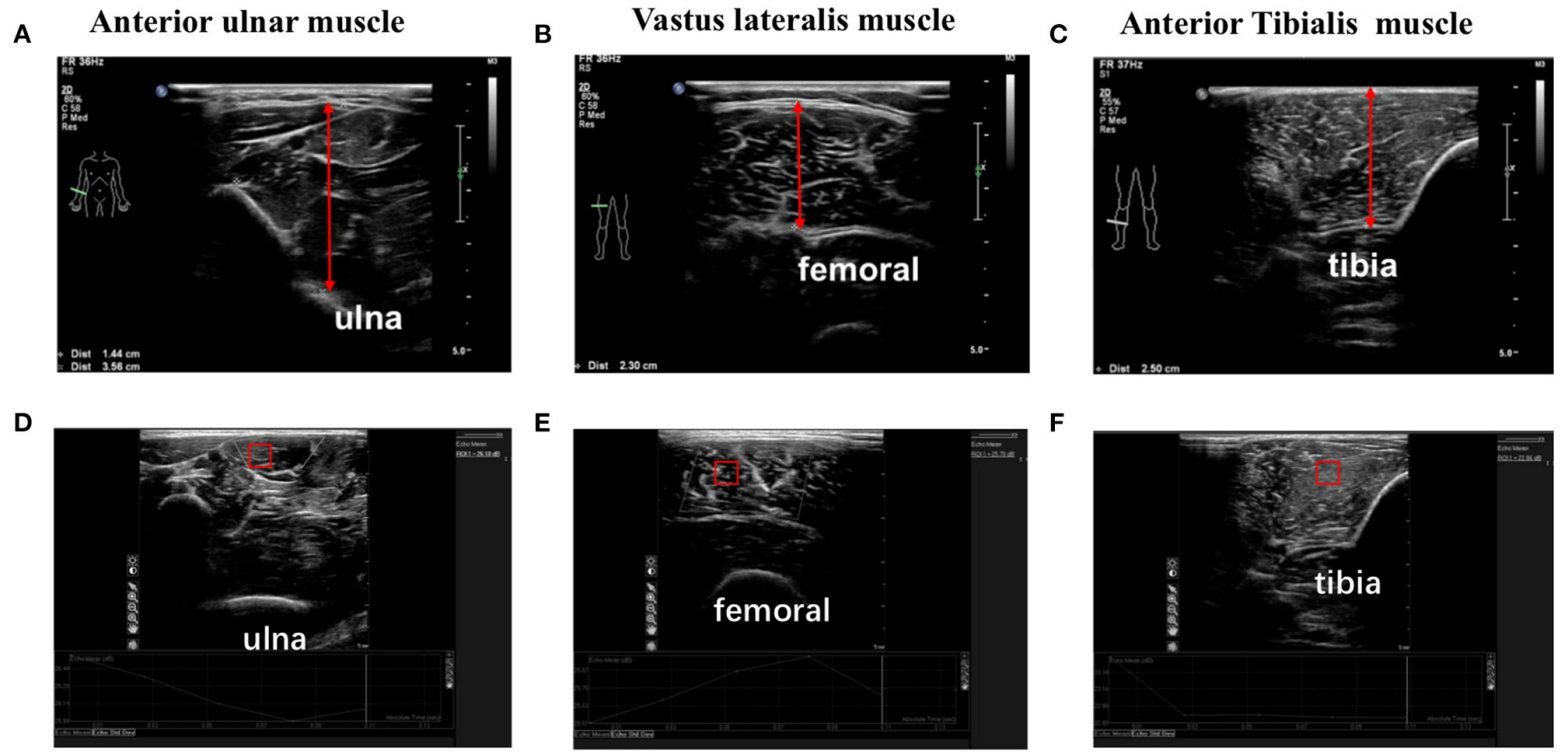
The thickness and quality of anterior ulnar muscle, vastus lateralis muscle and anterior tibialis muscle were detected by ultrasound. **(A–C)** The thickness of anterior ulnar muscle, vastus lateralis muscle, and anterior tibialis muscle were detected by ultrasound; **(D–F)** The gray value of anterior ulnar muscle, vastus lateralis muscle and anterior tibialis muscle were analyzed by QLAB software to detect the muscle quality. Square sampling frame represents Region Of Interest (ROI).

All patients underwent ultrasonic detection of the thickness of the anterior ulnar muscle (Figure 1A), the vastus lateralis muscle (Figure 1B) and the anterior tibial muscle (Figure 1C), and placed the 5 mm square sampling frame in the corresponding intramuscular image and obtain the Region Of Interest (ROI). The gray value was analyzed by QLAB software to represent muscle quality (Figures 1D–F).

As shown in Table 2, at the stage of prefrailty, the muscle thickness of the three parts did not change significantly compared with the normal group, but the grayscale value of the vastus lateralis muscle increased significantly, indicating the deterioration of muscle quality. At the frailty stage, the muscle thickness and quality of the vastus lateralis muscle and the anterior tibialis muscle of the lower extremities decreased significantly compared with the normal group and the prefrailty stage, but the muscle thickness of the anterior tibialis muscle of the upper extremities did not change, only the muscle quality decreased significantly compared with the normal group.

**Table 2:** Local muscle thickness and muscle quality in control group, Prefrailty group and Frailty group.

	**Normal**	**Prefrailty**	**Frailty**
**Anterior ulnar muscle**
MT(cm)	3.44 ± 0.11	3.52 ± 0.55	3.13 ± 0.39
Grayscale value	21.05 ± 2.83	19.57 ± 4.05	22.90 ± 4.43^#^
**Vastus lateralis muscle**
MT(cm)	1.55 ± 0.36	1.59 ± 0.36	1.28 ± 0.32^**^^##^
Grayscale value	25.11 ± 5.40	27.30 ± 3.50^*^	30.52 ± 6.37^**^^##^
**Anterior Tibialis muscle**
MT(cm)	2.62 ± 0.27	2.56 ± 0.36	2.42 ± 0.38^**^^##^
Grayscale value	29.53 ± 3.92	28.47 ± 5.95	31.14 ± 3.58^**^^##^

*compared with normal;

# *compared with prefrailty*.

*MT, muscle thickness*

* *P < 0.05*;

** *and*

## *P < 0.01*.

### Correlation Between FFP and Local Muscle Thickness and Muscle Quality

**Figure 2:**
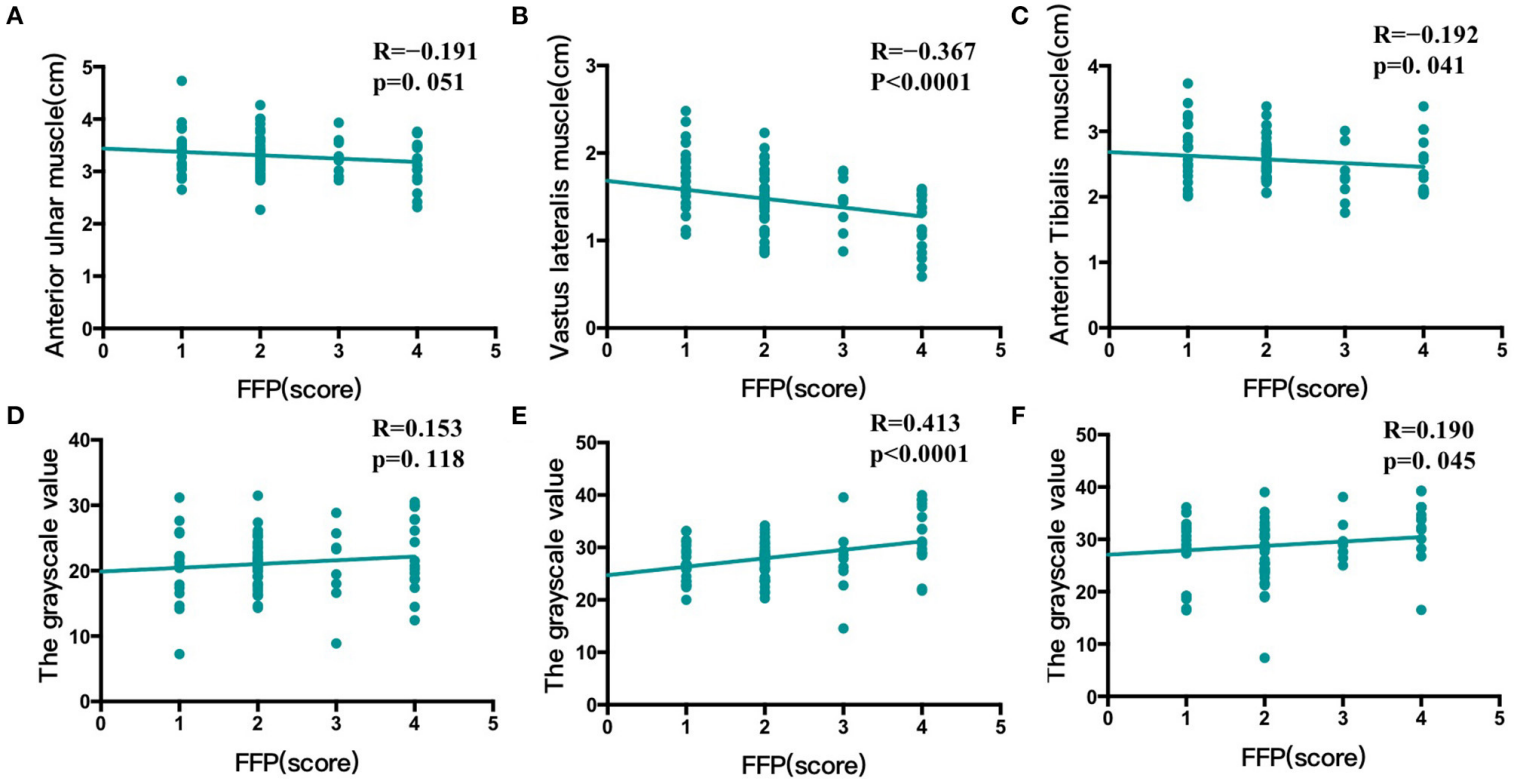
Correlation between FFP and muscle thickness and quality of anterior ulnar muscle, vastus lateralis muscle and anterior tibialis muscle. **(A)** There was no correlation between fried frailty phenotype (FFP) and the thickness of anterior ulnar muscle **(B)** FFP was negatively correlated with the thickness of vastus lateralis muscle **(C)** FFP was negatively correlated with the thickness of anterior tibialis muscle **(D)** There was no correlation between FFP and the gray value of anterior ulnar muscle **(E)** FFP was positively correlated with the gray value of vastus lateralis muscle **(F)** FFP was positively correlated with the gray value of anterior tibialis muscle.

There was no correlation between FFP and anterior ulnar muscle thickness and muscle quality (Figures 2A,D), however, it was negatively correlated with muscle thickness and muscle quality of the lower limbs, especially vastus lateralis muscle. As shown in Figure 2, with the increase of FFP, the thickness of vastus lateralis muscle decreased (*R* = −0.367, *P* < 0.0001) and the gray value increased (*R* = 0.413, *p* < 0.0001) (Figures 2B,E). There was only weak correlation between FFP and anterior tibial muscle (thickness: *R* = −0.192, *p* = 0. 041; gray value: *R* = 0.190, *p* = 0. 045) (Figures 2C,F).

### Multiple Logistics Regression Analysis of FFP, Local Muscle Thickness and Muscle Quality

Given the significant relationships between age, sex, BMI, glucose, Vitamin D, thyroid function and lipid metabolism and frailty, with FFP as the dependent variable and meaningful variables of univariate analysis as independent variables, multivariate logistics regression analysis was conducted (Table 3). The results indicated that FFP was positively associated with the gray value (Vastus lateralis muscle:β = 0.158, *p* = 0.021;Anterior Tibialis muscle:β = 0.107, *p* = 0.042) in the prefrailty stage, while positively associated with gray value (Vastus lateralis muscle:β = 0.457, *p* < 0.001; Anterior tibialis muscle: β = 0.220, *p* = 0.037) and inversely associated with muscle thickness (Vastus lateralis muscle:β = −0.973, *p* = 0.031; Anterior tibialis muscle: β = −4.551, *p* = 0.004) in the frailty stage. However, no correlation was found between FFP and muscle thickness and quality of anterior ulnar muscle. Moreover, in the frailty stage, FFP was positively associated with age (β = 0.164, *p* = 0.019) and negatively correlated with vitamin D (β = −0.231, *p* = 0.036) and FT3 (β = −0.342, *p* = 0.023).

**Table 3:** Multivariate logistics regression analysis of the effects of muscle thickness and muscle quality of Anterior ulnar muscle, Vastus lateralis muscle and Anterior Tibialis muscle on FFP.

	**Frailty**				**Prefrailty**			
	**β**	**Wardχ^2^**	***p*-value**	**OR(95%CI)**	**β**	**Wardχ^2^**	***p*-value**	**OR(95%CI)**
Age	0.164	5.493	**0.019**	1.179 (1.027, 1.352)	0.052	1.467	0.226	1.054 (0.968, 1.146)
BMI	0.032	0.056	0.814	1.033 (0.791, 1.347)	−0.055	0.431	0.511	0.946 (0.802, 1.116)
HbA1c	0.683	2.432	0.119	1.980 (0.839, 4.670)	0.485	2.549	0.110	1.623 (0.896, 2.943)
Glucose	−0.632	3.361	0.067	0.532 (0.271, 1.045)	−0.498	3.630	0.057	0.608 (0.364, 1.014)
VD	−0.231	4.380	**0.036**	0.971 (0.929, 1.015)	−0.027	1.704	0.192	0.974 (0.949, 0.998)
FT3	−0.342	0.182	**0.023**	0.711 (0.148, 3.414)	−0.262	0.728	0.394	0.769 (0.421, 1.405)
Depression	0.105	1.405	0.236	1.111 (0.934, 1.321)	0.066	1.435	0.231	1.068 (0.959, 1.190)
**Anterior ulnar muscle**
MT	0.716	0.240	0.624	2.046 (0.116, 35.985)	0.138	0.044	0.834	1.148 (0.316, 4.164)
Grayscale value	0.074	0.341	0.559	1.077 (0.839, 1.383)	−0.068	1.017	0.313	0.934 (0.819, 1.066)
**Vastus lateralis muscle**
MT	−0.973	0.296	**0.031**	0.378 (0.011, 12.555)	1.348	1.872	0.171	3.851 (558, 26.575)
Grayscale value	0.457	15.233	**0.000**	1.580 (1.256, 1.988)	0.158	5.344	**0.021**	1.171 (1.024, 1.340)
**Anterior Tibialis muscle**
MT	−4.551	8.084	**0.004**	0.011 (0.000, 0.243)	−1.700	4.143	0.062	0.183 (0.036, 0.939)
Grayscale value	0.220	4.328	**0.037**	1.246 (1.013, 1.532)	0.107	3.470	**0.042**	1.113 (0.994, 1.246)

*Fried Frailty Phenotype (FFP) as the dependent variable and meaningful variables of univariate analysis as independent variables. Adjustment for age, BMI, HbA1c, Glucose, VD, FT3 and Depression; β standardized coefficient. Bold values are statistically significant (p < 0.05)*.

## Discussion

In our study, ultrasonic measurements of the thickness and grayscale of the anterior ulnar muscle of the upper extremity and the vastus lateralis and anterior tibialis muscle of the lower extremity were performed on people over 65 years of age.

Participants in the prefrailty group had a higher grayscale value of the vastus lateralis muscle, indicating the deterioration of muscle quality. At the frailty stage, the muscle thickness and quality of the vastus lateralis muscle and the anterior tibialis muscle decreased significantly compared with the normal and the prefrailty group.

Pearson's correlation analysis showed no correlation between FFP and anterior ulnar muscle thickness and quality, however, it was negatively correlated with muscle thickness and quality of the lower limbs. These results suggested that the FFP is closely related to the thickness and quality of lower limb muscles, especially vastus lateralis muscle.

The use of ultrasound technology has gradually extended from the initial cardiovascular diseases to musculoskeletal diseases in recent years. With the development of muscle ultrasound, it has been known that sarcopenia declines at inconsistent rates in different areas of the body. With aging, the abdominal muscles decline first, followed by the lower limbs, and finally the upper limbs. The muscle decline rate of the lower limbs is faster than that of the upper limbs; Sometimes the upper limb muscle mass of the elderly will even increase due to compensation. Considering that the nutritional status of the elderly people included in this study is basically normal, the degree of sarcopenia may not be serious. These may be the reason why there was no correlation between the anterior ulnar muscle and FFP and weak relation between anterior tibialis muscle and FFP (15). Longitudinal studies of follow-up and animal models may be needed to explore the deeper factors such as gene and mitochondrial function and so on in the future. Also, it has been reported that local muscle changes can improve the diagnosis of sarcopenia compared with systemic muscle (16). As we all know, sarcopenia is the core of frailty. Thus, the analysis of muscle changes in different parts of the body can not only detect sarcopenia in the early stage but also exercise the local muscles with a specific aim to improve the local muscle function, which is of great significance to improve frailty, prevent disability, maintain the health of the elderly and improve the quality of life.

At present, the muscle thickness measured by ultrasound has a good correlation with the muscle mass measured by dual-energy X-ray absorption as the gold standard (17, 18). More importantly, the advantage of ultrasound lies not only in the evaluation of muscle mass in various parts of the body but also in the accurate detection of muscle quality (19). Muscle quality, defined as muscle unit cross-sectional area of muscle strength, is closely related to muscle function and is becoming a prominent factor affecting physiological function in the elderly (20). With aging, muscle fibers become thinner, connective tissue increases, lipid droplets infiltrate, extracellular water increases, and protein breakdown increases. These changes suggest poor muscle quality. MRI and CT have been used to assess muscle quality by determining fat infiltration into muscle and utilizing muscle attenuation (21). Echo-intensity measured by ultrasound can indicate lipid droplets infiltration and fibrosis in non-contractile tissues (22). Based on this information, the muscle quality was judged. The greater the value of ultrasonic echo intensity, the worse the muscle quality was suggested. Muscle quality is closely related to muscle strength and function and is a reliable indicator for early reflection of sarcopenia. In recent years, more and more studies have confirmed that the muscle thickness measured by ultrasound is closely related to muscle mass and strength, and it shows potential as a screening tool for frailty in older adults (23). Furthermore, in this study, we found that muscle thickness of the upper limb was not associated with FFP, while muscle thickness and quality of the vastus lateralis muscle were closely associated with FFP, and the muscle quality has deteriorated in the stage of prefrailty, which is earlier than the change of muscle thickness.

The pathogenesis of frailty is complicated, including genes and races, decreased exercise of the elderly, decreased protein intake and synthesis, changes in hormone levels, immunity, cell apoptosis and changes in the microenvironment, impaired mitochondrial function, and mental and psychological factors (24–27). Therefore, we have also conducted some clinical indicators, depression and frailty studies, then we found that in the frailty stage, blood glucose increased, vitamin D, FT3 levels significantly decreased, and depression scores increased, but had nothing to do with lipid. These results suggested that vitamin D supplementation is necessary for the elderly, thyroxine supplementation is necessary for patients with hypothyroidism, and psychological counseling is carried out to relieve depression.

There were certain limitations to this study. For example, since the better physical function is associated with improvement of frailty, we believe that being able to add physical activity is beneficial to analyze the risk factors for frailty. We will acquire data on activity levels, such as with the International Physical Activity Questionnaire (IPAQ). Second, due to the relatively high sample size, we got a small correlation between FFP and muscle thickness and quality of anterior tibialis muscle, more evidence will be needed to confirm this correlation. Finally, another limitation was the lack of interventions and follow-up due to their time-consuming nature. These shortcomings merit further study.

In conclusion, our study demonstrated that muscle quality has deteriorated in the stage of prefrailty, which is earlier than the decrease of muscle thickness. Moreover, FFP was closely related to local muscle thickness and quality, especially vastus lateralis muscle of the lower limbs. Together, our findings highlight the significance and practicability of ultrasound examination of local muscle with frailty assessment.

## Data Availability Statement

The original contributions presented in the study are included in the article/supplementary material, further inquiries can be directed to the corresponding authors.

## Ethics Statement

The studies involving human participants were reviewed and approved by Ethics Committee of The First Affiliated Hospital of Nanjing Medical University (No. 2019-SR-481). The patients/participants provided their written informed consent to participate in this study.

## Author Contributions

SL, LL, and HS: conceived the study, analyzed the data, and wrote the manuscript. XC, SC, SZ, and WL: subject recruitment and data collections. RL and GD: helped develop the design, gave critical input on interpretation of the results, and critically revised the manuscript draft. All authors contributed to the writing of the final manuscript draft and approved the version to be published.

## Funding

The study was funded by the National Natural Science Foundation of China (81871096), Health Research Project of Jiangsu Province (BJ20017), and the Opening Foundation (JSHD2021002).

## Conflict of Interest

The authors declare that the research was conducted in the absence of any commercial or financial relationships that could be construed as a potential conflict of interest.

## Publisher's Note

All claims expressed in this article are solely those of the authors and do not necessarily represent those of their affiliated organizations, or those of the publisher, the editors and the reviewers. Any product that may be evaluated in this article, or claim that may be made by its manufacturer, is not guaranteed or endorsed by the publisher.
